# University teachers’ beliefs about the use of generative artificial intelligence for teaching and learning

**DOI:** 10.3389/fpsyg.2024.1468900

**Published:** 2024-12-17

**Authors:** Beatriz Cabellos, Carlos de Aldama, Juan-Ignacio Pozo

**Affiliations:** ^1^Department of Psychology, Faculty of Health Sciences of King Juan Carlos University, Alcorcón, Spain; ^2^Division of Psychology of Higher Education Center Cardenal Cisneros, Madrid, Spain; ^3^Department of Research and Educational Psychology, Faculty of Education-Teacher Training Centre of the Complutense University of Madrid, Madrid, Spain; ^4^Department of Basic Psychology, Faculty of Psychology of the Autonomous University of Madrid, Madrid, Spain

**Keywords:** generative artificial intelligence, teacher beliefs, use of GenAI, pedagogical beliefs, teachers practices

## Abstract

**Introduction:**

The growing presence of generative artificial intelligence (GenAI) in our society, particularly in the educational field, is undeniable. This fact has led to various studies on its implications for learning and teaching. However, as with other technological resources, these implications will depend on how teachers use GenAI. Therefore, it is essential to identify teachers’ beliefs regarding the use of GenAI for teaching and learning.

**Methods:**

To this end, a questionnaire was designed and completed by 321 university teachers. This questionnaire consisted of two parts. The first included questions about the participants’ demographic information and a Likert scale on teachers’ pedagogical beliefs. The second part consisted of a 32-item Likert scale that evaluated teachers’ beliefs about the impact of GenAI on their students’ learning and their own teaching. These aspects were reflected through items that considered GenAI as either an educational opportunity or a threat.

**Results:**

The results showed that, of all the variables analyzed, only pedagogical beliefs and the frequency of previous GenAI use influenced beliefs about GenAI usage. Specifically, teachers with constructivist beliefs saw greater potential in GenAI compared to others. Similarly, teachers who regularly used these technologies had more positive beliefs about their educational use than those who used them sporadically or not at all. Lastly, it was also observed that while teachers valued the positive effects of GenAI on their teaching work, they also considered that its use could be detrimental to the learning processes of their students, making them more superficial.

**Discussion:**

These findings underline the importance of providing teachers with training focused on constructive approaches that enable them to maximize the potential of GenAI in education. In particular, it is crucial to promote teaching practices that, through student-centered GenAI use, foster active and reflective processes in students, aligned with the competencies demanded by today’s society.

## 1 Introduction

Generative Artificial Intelligence (GenAI) can be defined as algorithms designed to create content such as text, code, images, videos, and audio based on the data they have been trained on. However, unlike conversational artificial intelligence, GenAI systems not only provide replies but also possess the capability to generate the content of those responses (Mannuru et al., 2023).

These capabilities of GenAI have translated into advancements in fields such as artistic innovation (Wingström et al., 2024), medicine (Topol, 2019), and support for people with disabilities through adaptive solutions (Bennett and Keyes, 2020). Nevertheless, concerns have also emerged regarding its ethical implications and societal impact. Among the most frequent criticisms are its potential use for spreading misinformation via bots (Chowdhury and Oredo, 2022; Yapo and Weiss, 2018), risks to privacy and security, such as identity theft (Elliot and Soifer, 2022), and its disruptive impact on the labor market (Manyika et al., 2017). In addition to these practical concerns, there are also criticisms of a more epistemic nature. These include inherent biases stemming from the training data, the inability to overcome the limitations imposed by such data, the lack of true creativity, and difficulties in interpreting or contextualizing responses across different situations (Ali et al., 2024).

In the specific field of education, the impact of GenAI has not gone unnoticed. The use of these tools to mediate teaching and learning processes has shown both positive and negative results in various areas and disciplines (Lo, 2023).

Specifically, regarding its effects on learning, some authors point out that GenAI can impoverish learning if students merely copy and paste information without questioning the sources, or if they encounter unreliable or outdated information that can confuse them (Al-Mughairi and Bhaskar, 2024; Lo, 2023; Rodrigues et al., 2024). For example, Nisar and Aslam (2023) evaluated the extent to which ChatGPT could promote self-directed learning. The responses produced by this tool, although accurate and relevant, did not provide references or information sources, questioning its reliability and validity (Lo, 2023). Similarly, excessive use of these technologies can reduce students’ information management skills, as these tools search, analyze, and organize data by themselves (Al-Mughairi and Bhaskar, 2024). For example, in a controlled experiment, Darvishi et al. (2024) found that in a sample of 1,625 university students, most tended to rely on AI suggestions rather than actively learning from them. Likewise, other studies indicate that excessive reliance on GenAI can negatively impact decision-making (Buçinca et al., 2023; Lai and Tan, 2019).

In contrast, other studies highlight the benefits of using GenAI for learning. For example, Moussa et al. (2024) emphasize that these tools can enhance understanding of the studied content and promote a more active and creative role, as their use involves posing questions about the content to be developed. Similarly, the potential of GenAI to promote critical thinking has also been highlighted (Yusuf et al., 2024). For instance, Muthmainnah et al. (2022) identified through a sample of 453 students who used GenAI that these tools favored students’ critical thinking as they allowed them to contrast information from multiple sources and integrate diverse perspectives.

It is also relevant to highlight the changes that may arise in assessment activities by incorporating the use of GenAI. In this regard, some authors question whether using ChatGPT or other GenAI tools to draft texts meets the traditional criteria for the final product to be considered original (Dobrin, 2023). Traditionally, plagiarism has been understood as appropriating others’ ideas without acknowledging their contribution. However, the fact that the written product is generated by AI, not a particular person, complicates the possibility of attributing that recognition to a specific author. Despite this, there is some consensus that excessive contribution from GenAI in drafting a text should be considered plagiarism. For example, Ibrahim et al. (2023) surveyed over 500 teachers from different countries on this issue, finding that around 70% believed that using ChatGPT should be treated as plagiarism. In this sense, the incorporation of GenAI can be considered a threat to traditional forms of assessment as it hinders detection of student-generated content as opposed to tool-generated content (Chen et al., 2020; Dobrin, 2023). That said, to address this problem, some authors suggest conducting traditional real-time assessments where the use of these resources is prohibited (Lo, 2023).

Furthermore, the challenges associated with using these tools in other non-evaluative academic tasks should be considered. These tools, by providing instant answers to any question (regardless of accuracy), can generate fear among teachers of being replaced, leading them to propose banning or limiting their use in classes (Empantallados, 2023).

However, the use of GenAI in the classroom can also be seen as an opportunity for teachers to promote reflective activities on issues related to privacy and information use, fostering new forms of teaching that transcend simple knowledge reproduction (Chen et al., 2020; Zekaj, 2023). Additionally, GenAI can personalize learning tasks through intelligent tutoring systems, adapting to students’ levels and favoring a process and planning-centered approach (Bond et al., 2024; González-Calatayud et al., 2021; Lindsay et al., 2023; Liu et al., 2023; Lo, 2023). In this sense, Pardos and Bhandari (2023) compared the effectiveness of two tutoring systems, one supervised by teachers and another developed by ChatGPT, in learning various algebra-related content. The authors found that 70% of the instructions produced by ChatGPT met the minimum required quality criteria and favored learning gains. This fact can undoubtedly simplify teachers’ tasks when personalizing teaching. However, the authors also highlighted that human supervision was significantly better.

At this point, it is important to note that we believe the opportunities and threats regarding the inclusion of GenAI in teaching and learning contexts are not independent of the educational contexts in which they are integrated. On the contrary, we think these beliefs about the opportunities and threats associated with GenAI will be deeply related to the approach of teaching practices where these tools are integrated.

Traditionally, the literature has distinguished two types of teaching practice approaches to promote learning (Biggs and Tang, 2007): a content-centered approach and a student-centered approach.

In content-centered practices, typical of a traditional teaching approach, the priority is the transmission of information from the teacher to the student. Therefore, knowledge acquisition is measured by how accurately students replicate the taught content. Various studies, such as those conducted by Biagi and Loi (2013), Mueller and Oppenheimer (2014), and the OECD (2015), which analyze the use of Information and Communication Technologies (ICT) with a content-centered approach, have identified that these resources do not promote greater learning compared to others. This represents an evident limitation for integrating ICT in general, but it is accentuated in the case of GenAI, as its ability to generate closed content poses a threat to the learning of other content facilitated by the teacher.

In contrast, student-centered practices are characterized by an approach where content is considered a means to develop competencies in the student, promoting skills such as searching, managing, and reflecting on knowledge rather than merely accumulating information. These possibilities have been verified in the use of ICT with a student-centered approach through studies such as those by Chauhan (2017), Comi et al. (2017), and Xie et al. (2018), which have identified benefits in using ICT in the classroom when adopting this type of practice. Therefore, from this approach, the inclusion of GenAI can be considered an opportunity to promote essential competencies for today’s society, such as critical thinking and creativity, while fostering more active and personalized learning.

Thus, we consider that from a student-centered teaching approach, integrating GenAI presents significant learning opportunities, while from a content-centered approach, these resources pose certain threats that will increase teachers’ skepticism regarding their use. To conclude, we believe that understanding teachers’ beliefs about the use of GenAI from both approaches, will be essential.

### 1.1 Teachers’ beliefs about the use of GenAI in teaching and learning

Beliefs can be defined as personal statements about what is considered true or false (Reid and Amanat Ali, 2020). According to Harvey (1986), beliefs are mental constructs based on experience that are considered true, valid, and credible. These beliefs include an evaluative and judgmental component, unlike knowledge which is based on objective facts. Beliefs therefore represent subjective assessments of acquired knowledge. They are often confused with attitudes, which are evaluative predispositions, whether positive or negative, that guide behavior (Reid and Amanat Ali, 2020). In any case, beliefs are fundamental for predicting people’s behavior and attitudes (Ajzen, 1991; Fishbein and Ajzen, 1977).

In this regard, teachers’ beliefs can encompass the role that the school should play in society, the role of teachers and students, the function of the curriculum, epistemological beliefs about the nature of knowledge, pedagogical beliefs about the nature of teaching and learning processes, or even those that would lead us to consider the benefits or problems of using certain digital resources or tools such as GenAI.

Specifically, beliefs about the use of electronic devices, in general, have been considered key to predicting certain attitudes that affect their acceptance and subsequent uses (see the Technology Acceptance Model by Davis, 1985, or the Unified Theory of Acceptance and Use of Technology by Venkatesh et al., 2003 for a more detailed analysis).

In the specific case of GenAI, similar results have been identified that demonstrate this relationship between attitudes and uses (Gursoy et al., 2019; Ofosu-Ampong, 2024). However, there are not many studies that delve into the specific beliefs of teachers about the benefits and risks of GenAI. In general, teachers seem to consider that GenAI can be beneficial for their teaching work (Moussa et al., 2024; Mustafa, 2024; Polak et al., 2022; Xue and Wang, 2022), although they are also aware of some problems arising from its use (Moussa et al., 2024; Mustafa, 2024; Empantallados, 2023; Shamsuddinova et al., 2024).

For example, Bewersdorff et al. (2023) conducted a review of the most frequent myths and misconceptions about AI. The authors concluded that, in most cases, users had a limited understanding of GenAI at a technical level, as well as an uninformed opinion about the scope, possibilities, and limitations of these tools. Similarly, Chounta et al. (2022) surveyed a sample of 140 teachers about their knowledge and concerns regarding the use of GenAI in educational contexts. Despite teachers perceiving GenAI as an opportunity for teaching and learning, the results revealed a limited understanding of how to implement these tools in the classroom. These conclusions are slightly different from those formulated by Antonenko and Abramowitz (2023). In this study, most teachers had accurate conceptions and identified erroneous statements about GenAI (e.g., GenAI is just a fad, something new, or something exclusive to technology companies). However, in many cases, teachers failed to recognize fundamental aspects for teaching and learning, such as detecting biases in the information provided by GenAI.

Thus, as a synthesis, we can conclude that some questions remain unanswered: To what extent do teachers believe that GenAI is an opportunity to promote new ways of teaching and learning? Or do they perceive it more as a threat that hinders traditional ways of understanding educational practice? Which GenAI possibilities are regarded as opportunities or threats? This paper aims to answer these questions.

Another unanswered question remains. What effect do certain variables have on beliefs about GenAI as an opportunity or a threat? To date, variables such as pedagogical beliefs, previous educational use of technologies, and sociodemographic factors such as gender, teaching experience, and area of knowledge also seem to affect beliefs about the use of digital tools. Will this effect be present in specific beliefs about GenAI in educational contexts? We will address this issue below.

### 1.2 What variables can affect beliefs about GenAI for teaching and learning?

#### 1.2.1 Pedagogical beliefs and their relationship with beliefs about GenAI for teaching and learning

As previously discussed, beliefs about GenAI as an opportunity or a threat cannot be understood independently of teaching practices since these beliefs are likely to be closely related to either a content-centered or student-centered teaching approach. Teaching practices do not occur randomly but are influenced by various aspects, such as teacher training, extrinsic barriers like curriculum rigidity or lack of institutional support, and intrinsic barriers, which include the more implicit beliefs that teachers hold about teaching and learning processes (Ertmer et al., 2015; de Aldama and Pozo, 2016; Liu, 2011). These intrinsic barriers, referred to as pedagogical beliefs, are the ones that will most significantly affect the adoption of one teaching practice approach over another (Fives and Gill, 2015; Hofer and Pintrich, 1997, 2002; Pozo et al., 2006). In this regard, the literature indicates that content-centered practices are based on reproductive pedagogical beliefs, also known as behaviorist by some authors (see, for example, Arancibia et al., 2020). These beliefs are based on the idea that learning is a faithful copy of the presented reality, achieved through associative processes without the intervention of psychological processes. According to this perspective, learning depends on both the task and the learner characteristics.

In contrast, student-centered practices are based on constructive pedagogical beliefs. This understanding of teaching and learning processes is characterized by not considering knowledge as fixed, but rather as transformed by the mental processes activated in the learner. The success of learning, therefore, depends on the teaching conditions activating certain mental processes so that students progressively take control of these processes (Pozo et al., 2021; Pozo et al., 2006).

However, from our position, teachers’ beliefs are more complex and less dichotomous than indicated (Ertmer et al., 2015). Research suggests that these beliefs form a continuum between the reproductive and constructive extremes (Hofer and Pintrich, 1997, 2002). This explains why some authors mention the existence of intermediate beliefs, known as interpretative beliefs about teaching and learning processes (Pérez Echeverría et al., 2001; Pozo et al., 2006). These interpretative beliefs consider learning as a precise reflection of reality, similar to reproductive beliefs, but also recognize that teaching is mediated by the student’s cognitive processes, leading to more varied practices closer to the constructive approach.

To conclude, we believe teachers’ pedagogical beliefs will partly underpin more student-centered or content-centered practices, which in turn will explain whether GenAI is perceived as an opportunity or a threat to teaching and learning processes.

Although no studies directly identify the relationship of pedagogical beliefs when considering beliefs about GenAI as an opportunity or a threat and how they integrate into teaching, Choi et al. (2023) studied how these beliefs affected teachers’ acceptance of GenAI. They found that teachers with constructive beliefs were more likely to integrate educational GenAI tools than those with more reproductive orientations.

These results align with other studies that have identified the effect of these pedagogical beliefs on the use of ICT, suggesting that they influence its use and integration in the classroom (Ertmer, 2005; Ertmer et al., 2015; Ertmer and Ottenbreit-Leftwich, 2010). Specifically, studies like those by Liu (2011), Liu et al. (2017), Sang et al. (2010), and Tondeur et al. (2017) found that constructive pedagogical beliefs promote more positive attitudes toward ICT and more student-centered use, in contrast to more reproductive beliefs that represent a barrier to effective ICT integration due to their predominantly content-centered focus.

#### 1.2.2 Previous educational use of GenAI and its relationship with beliefs about GenAI for teaching and learning

Previous educational use of GenAI can be understood by the frequency with which teachers use these resources to carry out various teaching activities.

In this regard, it is likely that this familiarity promotes an understanding of the possibilities of tools like GenAI. Although the literature in this area is incipient, some studies have attemptedto identify the effect of this familiarity on beliefs about GenAI. For example, Kaplan-Rakowski et al. (2023) asked 147 teachers, mostly university teachers, about their use of GenAI in educational contexts. The results showed that those who used GenAI more frequently had a more positive opinion. Other studies focused on teaching experience with the general use of ICT showed that teachers who were more familiar with digital technologies were more willing to experiment with GenAI and integrate it into their teaching practices (Fietta et al., 2022; Schepman and Rodway, 2020).

Finally, similar results have been found in the specific use of ICT. For example, some research has shown that teachers who use ICT more frequently have more positive beliefs and more varied and frequent uses of these resources (Cabellos et al., 2023; Drent and Meelissen, 2008; Pozo et al., 2021).

#### 1.2.3 Sociodemographic variables and their relationship with beliefs about GenAI for teaching and learning

As previously mentioned, certain sociodemographic variables of teachers, such as gender, teaching experience, or the area of teaching, have commonly been studied due to their effect on teachers’ beliefs about the general use of ICT. Specifically, in the case of GenAI, it has been identified that women have more cautious beliefs toward GenAI compared to men, who tend to show greater openness and enthusiasm (Albarrán-Lozano et al., 2021; Fietta et al., 2022). Similar results have also been observed in the general use of ICT (Li and Kirkup, 2007; Drabowicz, 2014; Mathews and Guarino, 2000; Cai et al., 2017). However, other studies (Gorder, 2008; Law and Chow, 2008; Pozo et al., 2021) have not found significant differences in this aspect or even found opposite results where women valued the benefits of ICT more (Guillén-Gámez and Rodríguez-Fernández, 2021).

On the other hand, although the effect of teachers’ experience or age on beliefs about GenAI has not been specifically investigated, studies on the adoption of ICT have found that the older the teachers, the less interest they have in ICT (Guillén-Gámez and Mayorga-Fernández, 2020; Suárez et al., 2012; Van Braak et al., 2004). However, other studies have not corroborated this relationship (Gaonkar, 2018; Gorder, 2008; Inan and Lowther, 2010; Law and Chow, 2008). Regarding teaching experience, the results are also contradictory; some studies have indicated a negative relationship, meaning more negative attitudes toward ICT as age or experience increases (Gaonkar, 2018; Mathews and Guarino, 2000; Baek et al., 2008; Inan and Lowther, 2010), while others have found no correlation (Gorder, 2008).

Lastly, regarding areas of knowledge, research suggests that teachers in STEM fields (Science, Technology, Engineering, and Mathematics) are more inclined to adopt GenAI due to the natural affinity of these disciplines with technology. In contrast, teachers in humanities and arts tend to be more reluctant, justified by the fact that these disciplines are less connected to such tools and therefore teachers are not familiar with their use in educational contexts, which would explain more skeptical beliefs (Ayanwale and Sanusi, 2023). This result contrasts with those obtained in the general case of ICT, where generally no differences have been identified based on the area of knowledge (Gorder, 2008; Pozo et al., 2021; Vanderlinde et al., 2010; Williams et al., 2000).

## 2 Research questions and objectives

Based on the above, as we have pointed out, it is necessary to delve deeper into whether teachers believe that the use of GenAI will pose a threat to the usual ways of learning and teaching or, rather, will provide an opportunity to transform these practices in classrooms, also checking which variables affect these beliefs. To do this, as already anticipated, this study asked teachers, specifically university teachers, to complete a questionnaire to identify their beliefs about the effects of using GenAI for teaching and learning. Do teachers believe that GenAI will promote more superficial learning and uncritical acceptance of information? Or, on the contrary, will it be a resource that will promote new, more personalized ways of learning and help develop critical thinking? Will it replace and impoverish teachers’ work? Or rather, will it promote an abandonment of traditional teaching roles to guide teaching toward more complex forms of dialog? Will it hinder true assessment of learning by making plagiarism easier? Or, on the contrary, will it promote new, more personalized forms of assessment, rather than limiting itself to evaluating the reproduction of acquired knowledge? Will these beliefs about GenAI be influenced by pedagogical beliefs, previous use of GenAI, or demographic factors? To answer these questions, we set the following objectives:

- Objective 1: Identify university teachers’ beliefs about the use of GenAI for teaching and learning, determining to what extent they consider GenAI an opportunity to improve teaching and learning or a threat to these processes.
- Objective 2: Analyze the variables that influence these beliefs (gender, teaching experience, area of knowledge, pedagogical beliefs, and previous educational use of GenAI).
- Objective 3: Identify university teachers’ beliefs about the use of GenAI in relation to student activities (“Student’s learning processes” and “Student’s information management”) and teacher activities (“Teacher’s role in assessment” and “Teacher’s role in teaching”). Specifically, determine to what extent they consider GenAI as an opportunity or a threat in relation to these dimensions.
- Objective 4: Analyze which variables can influence these beliefs about student and teacher activities (gender, teaching experience, area of knowledge, pedagogical beliefs, and previous educational use of GenAI).

## 3 Method

### 3.1 Participants

The participants of the study were teachers from various public and private universities in Spain, belonging to different areas of knowledge. To access them, we used university email directories. To incentivize participation, we raffled off 75 euros for the purchase of teaching materials among all participants. We collected 332 responses between March and April 2024, from which we eliminated 11 participants who completed the questionnaire in less than 5 min, an insufficient time to read and complete the questionnaire adequately. Therefore, the final sample consisted of 321 university teachers (see Table 1).

**Table 1:** Characteristics of the sample and variables.

		Frequency	Percentage
Gender*	Man	152	47.40
	Woman	168	52.30
Teaching experience	Less than 10 years	109	34.00
	From 11 to 20 years	87	27.10
	More than 20 years	125	38.90
Area of knowledge	Sciences (e.g., Mathematics, Physics)	32	10.00
	Health Sciences (e.g., Biology, Environmental Sciences)	41	12.80
	Engineering and Architecture (e.g., Industrial Engineering, Architecture)	34	10.60
	Social Sciences and Law (e.g., Psychology, Law)	151	47.00
	Arts and Humanities (e.g., Art History, Philology)	63	19.60
Previous educational use of GenAI	Never	131	40.80
	Sometimes	151	47.00
	Frequently	39	12.10
Pedagogical beliefs	Reproductive	75	23.40
	Interpretative	186	57.90
	Constructive	60	18.70

*One participant who identified as non-binary was treated as missing data in the gender variable analysis due to insufficient sample size.

### 3.2 Instrument

Data collection was conducted through a questionnaire divided into two sections. In the first section, after participants gave their informed consent, they were asked for personal (gender) and professional information (teaching experience, area of knowledge, pedagogical beliefs, and previous educational use of GenAI). They were also asked to complete the 6-point Likert scale on pedagogical beliefs by De Vries et al. (2014) in its Spanish validated version by Arancibia et al. (2020) (see Appendix 1). This scale evaluated the importance attributed to different educational activities, classified as behaviorist (renamed in this study as reproductive) or constructive, where 1 referred to “not important at all” and 6 “very important.”

The second section focused on identifying university teachers’ beliefs about the use of GenAI in teaching and learning. For this purpose, we developed a Likert scale in which participants were asked to express their degree of agreement with various statements about the possible opportunities or threats of GenAI in teaching and learning. This scale had 6 points, where 1 implied “strongly disagree” and 6 “strongly agree.”

The items were distributed across four main dimensions, two focused on how students learn (“Student’s learning processes” and “Student’s information management”) and two focused on teachers (“Teacher’s role in assessment” and “Teacher’s role in teaching”). Each of these was subdivided into items that either reflected the opportunities of GenAI to promote new forms of teaching and learning or referenced GenAI as a threat to traditional forms of teaching and learning.

To ensure the validity of this scale, an inter-rater task was carried out in which 13 experts participated in identifying the different items formulated in each dimension. From this task, 4 items were eliminated, and the wording of the most problematic ones was modified. Finally, the scale consisted of 32 items.

Table 2 presents a detailed description of each dimension with an example of the corresponding items. Additionally, Appendix 2 contains the complete questionnaire items within their respective dimensions.

**Table 2:** Structure and examples of questionnaire items.

Dimensions	Definition	To what extent do you believe GenAI can…	
		Item on the use of generative AI seen as an opportunity	Item on the use of generative AI seen as a threat
Student’s learning processes	This dimension evaluates how generative AI influences the quality of student learning, their active participation in the educational process, and their ability to understand and create content. Emphasis is placed on how generative AI can promote deeper, active, and creative learning, but also on how it can harm by fostering superficial and passive learning.	…promote deeper learning by helping students better understand the content	…limit students’ creativity by generating finished answers to the requested topic.
Student’s information management	This dimension focuses on how students handle and evaluate the information obtained through generative AI. Emphasis is placed on generative AI’s ability to promote critical thinking and privacy awareness, but also on the risks associated with unreliable information and the lack of personal elaboration.	…encourage students to learn to generate better questions for generative AI to seek information.	…encourage students to copy and paste information without questioning the sources.
Teacher’s role in assessment	This dimension addresses the impact of generative AI on the strategies and methods of assessment used by teachers. Emphasis is placed on how generative AI can promote new forms of evaluation and feedback management, but also on the challenges it presents for detecting plagiarism and assessing authentic performance.	…promote new forms of evaluation beyond mere reproduction of knowledge.	…make it difficult for teachers to evaluate how students arrived at their answers.
Teacher’s role in teaching	This dimension focuses on the impact of generative AI on the daily tasks of teachers, including how it affects the planning and execution of educational activities. Emphasis is placed on how generative AI can help tailor activities to students’ levels and interests and manage knowledge, but also on the challenges to maintain the relevance of teachers and the quality of academic activities.	…allow teachers to tailor activities to the levels and interests of students.	…harm academic activities, making it advisable to prohibit or restrict its use as much as possible.

### 3.3 Data analysis

The reliability of the questionnaire on beliefs about the use of GenAI in teaching and learning was evaluated with the scales relating to the opportunities of GenAI and separately with those referring to the threats, given that their contents were opposed. For this, we used the Omega coefficient. It was found that the reliability of the total items, both in those referencing opportunities for using GenAI and those highlighting threats, was above 0.90, indicating excellent internal consistency. Additionally, the subdimensions of the questionnaire presented reliabilities above 0.70, except in the “Teacher’s role in teaching” subdimension for items highlighting the risks of using GenAI, where reliability was 0.69. Despite being slightly below the desired threshold of 0.70, it was considered acceptable for the study’s purposes due to its proximity to the cutoff value.

The demographic variables collected in the questionnaire (gender, teaching experience, area of knowledge, and previous educational use of GenAI) were grouped into fewer levels than those established in the questionnaire to ensure appropriate statistical treatment of the data. Additionally, the pedagogical beliefs obtained from the De Vries et al. (2014) questionnaire were also coded as categorical variables to facilitate analysis. To establish these categories, the difference between constructive and reproductive items was calculated. Based on this difference, a categorical variable with three levels was created, based on the categorization seen in the introduction of this work:

- Values lower than −0.75 were classified as reproductive.
- Values between −0.75 and + 0.75 were classified as interpretative.
- Values higher than +0.75 were classified as constructive.

To analyze beliefs about the use of GenAI for teaching and learning, the mean of items understood as opportunities and threats was calculated separately for each dimension of the questionnaire as well as for the general items. Additionally, differential variables were created between beliefs as opportunities and as threats. Positive values, greater than 0, highlighted a tendency toward beliefs about GenAI as an opportunity, in contrast to negative values, below 0, which emphasized the threats toward GenAI. These differences provided a net measure of beliefs, facilitating the interpretation and analysis of the data.

For Objective 1 of the study, *identifying university teachers’ beliefs about the use of GenAI for teaching and learning*, the corresponding means and standard deviations were calculated. Additionally, for Objective 2, *analyzing which* var*iables influence these beliefs*, one-way ANOVA analyses were used. Significant differences between groups defined by categorical variables were therefore able to be assessed.

At this point, it is important to note that the variable pedagogical beliefs could be related to the previous use of GenAI, which should be controlled when analyzing the effect of previous GenAI use on beliefs about these resources. For this, a Chi-Square test of independence was conducted between these two variables, which were ultimately not related (*p* > 0.05), so controlling for pedagogical beliefs in the previous educational use of GenAI was deemed unnecessary.

Objective 3, *identifying university teachers’ beliefs about the use of GenAI in relation to student and teacher activities*, was carried out using a single-factor repeated measures ANOVA, which allowed for comparing the different dimensions of beliefs about the use of GenAI. Finally, to address Objective 4, *analyzing which* var*iables influence these beliefs about possible student and teacher activities*, two-factor ANOVAs were implemented, where one factor was completely randomized, and the other was repeated measures. The completely randomized factor allowed for comparing groups by their categorical variables, and the repeated measures factor identified differences between beliefs in terms of perceived opportunities and threats toward GenAI.

In all ANOVA analyses, *post hoc* tests were conducted to obtain a more detailed understanding of the differences between groups and experimental conditions. Additionally, in the two-factor ANOVAs, the effect of interaction was also analyzed. All analyses were carried out using SPSS statistical software, version 28.

## 4 Results

### 4.1 What beliefs did university teachers have about the use of GenAI for teaching and learning?

Regarding Objective 1, which consisted of *identifying university* teacher*s’ beliefs about the use of GenAI for teaching and learning*, it was observed that teachers considered the use of GenAI both as an opportunity (*M* = 3.96, *SD* = 0.87) and as a threat (*M* = 3.87, *SD* = 0.90) with similar frequency (*DM*^1^ = 0.09, *SD* = 1.48).

### 4.2 What variables influenced university teachers’ beliefs about the use of GenAI for teaching and learning?

Although there were no differences between the perception of GenAI as an opportunity or as a threat for teaching, when *analyzing the influence of the studied variables* (Objective 2), differences were found among teachers based on their pedagogical beliefs (*F* = 9.13, *p* < 0.001, *η^2^* = 0.05) and previous educational use of GenAI (*F* = 45.72, *p* < 0.001, *η^2^* = 0.22) at all levels of the variables (*p* < 0.001).

**Figure 1:**
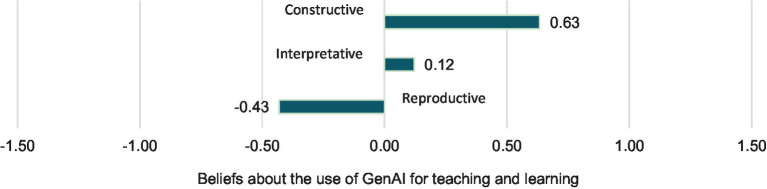
The effect of pedagogical beliefs on beliefs about the use of GenAI for teaching and learning.

Specifically, it was found (see Figure 1) that teachers with a reproductive orientation valued the use of GenAI in teaching less, considering it more of a threat in learning and teaching activities (*DM* = −0.43, *SD* = 1.54), followed by those with an interpretative orientation (*DM* = 0.12, *SD* = 1.42). In contrast, teachers with a constructive orientation had the most positive beliefs (*DM* = 0.63, *SD* = 1.39), emphasizing its use as an opportunity to promote new learning and teaching activities.

**Figure 2:**
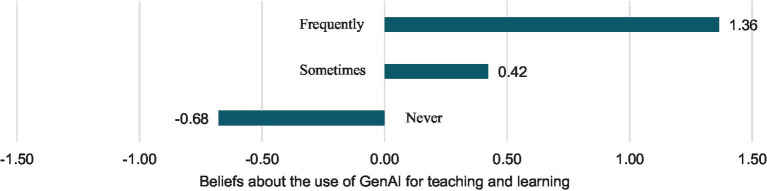
The effect of previous educational use of GenAI on beliefs about the use of GenAI for teaching and learning.

Additionally, regarding the previous educational use of GenAI (see Figure 2), it was observed that those who had never used GenAI perceived its use more as a threat (*DM* = −0.68, *SD* = 1.51). On the other hand, those who had used it occasionally (difference of means = 0.42, *SD* = 1.19) and especially teachers who used it more frequently highlighted the opportunities these resources offer for teaching (*DM* = 1.36, *SD* = 0.99).

The rest of the studied variables, including gender, teaching experience, or the area of knowledge in which they taught, did not affect teachers’ beliefs about the use of GenAI in teaching.

### 4.3 What beliefs did university teachers have about the use of GenAI regarding its effect on specific aspects of teaching and learning?

**Figure 3:**
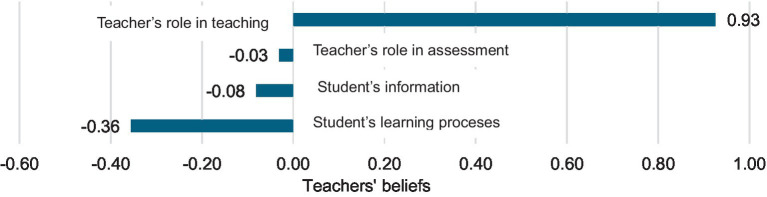
Difference of means among the different dimensions of the questionnaire.

**Figure 4:**
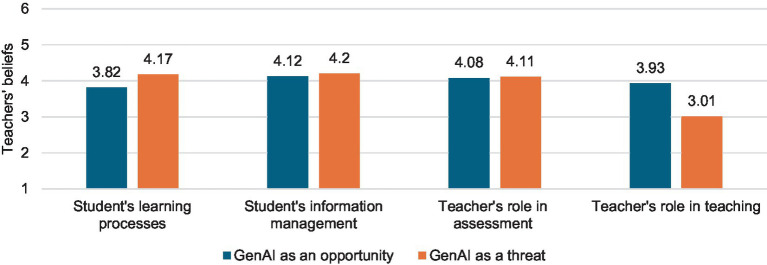
Differences between teachers’ beliefs about GenAI as an opportunity or threat across different dimensions of the questionnaire.

As previously noted, there were no overall differences in teachers’ beliefs about GenAI as an opportunity or threat in teaching and learning. However, when we closely *examined the different dimensions of beliefs about GenAI, referring to student and teacher activities* (Objective 3), the pattern became more complex (see Table 2). Specifically, significant differences were found (*F* = 177.61, *p* < 0.001, *η^2^* = 0.36) among all dimensions, except for “Student’s information management” and “Teacher’s role in assessment,” where the opportunities and threats of using GenAI were considered equivalently (see Figure 3). An especially optimistic stance was observed in “Teacher’s role in teaching” (see Figure 3) with particularly large differences (*p* < 0.001, *η^2^* > 0.40) when compared with the other three dimensions. This was because teachers believed that, regarding this dimension, GenAI was more of an opportunity (*M* = 3.93, *SD* = 0.96) than a threat (*M* = 3.01, *SD* = 0.96) (*F* = 119.22, *p* < 0.001, *η^2^* = 0.27) (see Figure 4).

In contrast, the dimension “Student’s learning processes” led to a more skeptical stance among teachers (see Figure 3). However, the effect sizes were more moderate compared to the other two dimensions (*p* < 0.001, *η^2^* > 0.80). Additionally, when distinguishing the opportunities and threats of GenAI in this dimension, it was observed that teachers saw GenAI more as a threat to “Student’s learning processes” (*M* = 4.17, *SD* = 1.10) than as an opportunity for improvement (*M* = 3.82, *SD* = 0.74) (*F* = 20.28, *p* < 0.001, *η^2^* = 0.06) (see Figure 4).

### 4.4 What variables influenced university teachers’ beliefs about the use of GenAI regarding its effect on specific aspects of teaching and learning?

To identify Objective 4, *which variables affected university* teacher*s’ beliefs about the use of GenAI in relation to student and teacher activities* (see Table 2), it was once again observed that neither gender, teaching experience, nor the area of knowledge affected beliefs about the use of GenAI. However, both pedagogical beliefs and previous use of GenAI did generate significant differences. Table 3 shows how these variables interacted with beliefs about GenAI as an opportunity and threat for each of the dimensions and where specifically these differences were found based on *post hoc* tests.

**Table 3:** Effect of pedagogical beliefs and previous educational use of GenAI on teachers’ beliefs about GenAI as an opportunity or threat across different dimensions of the questionnaire.

		Interaction	*Post hoc* (differences between levels of the IV within the dimension)*	*Post hoc* (differences between GenAi as an opportunity and threat dimensions within the level of the IV)
Student’s Learning Processes	Pedagogical beliefs	*F* = 3.43, *p* < 0.05, *η^2^* = 0.02	*GenAI as an **opportunity*** Reproductive-**Interpretative** (*DM* = −0.28, *p* < 0.05) Reproductive-**Constructive** (*DM* = −0.32, *p* < 0.05) Interpretative-Constructive (no difference) *GenAI as a **threat*** (no difference)	**Reproductive (threat)** (*DM* = −0.67, *p* < 0.001) **Interpretative (threat)** (*DM* = −0.33, *p* < 0.01) Constructive (no difference)
	Previous Educational USe of GenAI	*F* = 34.30, *p* = < 0.001, *η^2^* = 0.18	*GenAI as an **opportunity*** Never-**Sometimes** (*DM* = −0.32, *p* < 0.001) Never-**Frequently** (*DM* = −0.53, *p* < 0.001) Sometimes-Frequently (no difference) *GenAI as a **threat*** **Never-**Sometimes (*DM* = 0.59, *p* < 0.001) **Never**-Frequently (*DM* = 1.23, *p* < 0.001) **Sometimes**-Frequently (*DM* = 0.64, *p* < 0.01)	**Never (−)** (*DM* = −1.00, *p* < 0.001) Sometimes (no difference) **Frequently (opportunity)** (*DM* = 0.76, *p* < 0.001)
Student’s Learning Processes	Pedagogical beliefs	*F* = 6.73, *p* < 0.001, *η^2^* = 0.04	*GenAI as an **opportunity*** Reproductive-**Interpretative** (*DM* = −0.49, *p* < 0.001) Reproductive-**Constructive** (*DM* = −0.81, *p* < 0.001) Interpretative- Constructive (no difference) ***GenAI as a threat*** (no difference)	**Reproductive (−)** (*DM* = −0.58, *p* < 0.001) Interpretative (no difference) **Constructive (opportunity)** (*DM* = 0.40, *p* < 0.05)
	Previous Educational Use of GenAI	*F* = 32.59, *p* = < 0.001, *η^2^* = 0.17	*GenAI as an **opportunity*** Never-**Sometimes** (*DM* = −0.70, *p* < 0.001) Never-**Frequently** (*DM* = −1.04, *p* < 0.001) Sometimes-Frequently (no difference) *GenAI as a **threat*** **Never**-Sometimes (*DM* = 0.26, *p* = < 0.05) **Never**-Frequently (*DM* = 0.92, *p* < 0.001) **Sometimes**-Frequently (*DM* = 0.65, *p* < 0.001)	**Never (−)** (*DM* = −77, *p* < 0.001) Sometimes (no difference) **Frequently (opportunity)** (*DM* = 1.18, *p* < 0.001)
Teacher’s Role in Assessment	Pedagogical beliefs	*F* = 10.82, *p* < 0.001, *η^2^* = 0.06	*GenAI as an **opportunity*** Reproductive-**Interpretative** (*DM* = −0.41, *p* < 0.001) Reproductive-**Constructive** (*DM* = −0.65, *p* < 0.001) Interpretative- Constructive (no difference) *GenAI as a **threat*** Reproductive-Interpretative (no difference) **Reproductive**-Constructive (*DM* = 0.06, *p* < 0.01) **Interpretative**-Constructive (*DM* = 0.04, *p* < 0.05)	**Reproductive (−)** (*DM* = −0.59, *p* < 0.001) Interpretative (no difference) **Constructive (opportunity)** (*DM* = 0.63, *p* < 0.001)
	Previous Educational Use of GenAI	*F* = 30.96, *p* < 0.001, *η*^2^ = 0.16	*GenAI as an **opportunity*** Never-**Sometimes** (*DM* = −0.51, *p* < 0.001) Never-**Frequently** (*DM* = −0.68, *p* < 0.001) Sometimes-Frequently (no difference) *GenAI as a **threat*** **Never**-Sometimes (*DM* = 0.47, *p* < 0.001) **Never**-Frequently (*DM* = 1.15, *p* < 0.001) **Sometimes**-Frequently (*DM* = 0.68, *p* < 0.001)	**Never (−)** (*DM* = −0.72, *p* < 0.001) **Sometimes (opportunity)** (*DM* = 0.27, *p* < 0.05) **Frequently (opportunity)** (*DM* = 1.12, *p* < 0.001)
Teacher’s Role in Teaching	Pedagogical beliefs	*F* = 12.10, *p* = < 0.001, η^2^ = 0.07	*GenAI as an **opportunity*** Reproductive-**Interpretative** (*DM* = −0.55, *p* < 0.001) Reproductive-**Constructive** (*DM* = −0.89, *p* < 0.001) Interpretative-**Constructive** (*DM* = −0.33, *p* < 0.05) *GenAI as a **threat*** (no difference)	Reproductive (no difference) **Interpretative (opportunity)** (*DM* = 0.97, *p* < 0.001) **Constructive (opportunity)** (*DM* = 0.1.56, *p* < 0.001)
	Previous Educational Use of GenAI	*F* = 43.38, *p* = < 0.001, η^2^ = 0.21	*GenAI as an **opportunity*** **Never**-Sometimes (*DM* = −0.67, *p* < 0.001) **Never**-Frequently (*DM* = −0.89, *p* < 0.001) Sometimes-Frequently (no difference) *GenAI as a **threat*** Never-**Sometimes** (*DM* = 0.54, *p* < 0.001) Never-**Frequently** (*DM* = 1.02, *p* < 0.001) Sometimes-**Frequently** (*DM* = 0.48, *p* < 0.01)	Never (no difference) **Sometimes (opportunity)** (*DM* = 1.33, *p* < 0.001) **Frequently (opportunity)** (*DM* = 2.04, *p* < 0.001)

*The direction of the differences can be observed through the negative and positive sign in the DM (Differences of Means). To facilitate result interpretation it was decided to highlight in bold the significant effects and their direction as either an opportunity or a threat according to pedagogical beliefs and Previous Educational Use of GenAI.

As shown in Table 3, in general terms, we found that pedagogical beliefs significantly influenced the differences between teachers’ beliefs about the use of GenAI as an opportunity or threat, indicating an interaction between both variables (*p* < 0.05), although with low effect sizes (*η^2^* < 0.07). Specifically, analyzing the dimensions where GenAI is conceived as an opportunity, teachers with reproductive beliefs scored lower than interpretative and constructive ones (*p* < 0.05). In the “Teacher’s role in teaching” dimension, interpretative teachers also attributed fewer possibilities to GenAI for learning and teaching than constructive teachers (*p* < 0.05). However, in the dimensions referring to the threats associated with using GenAI in teaching and learning, differences were only found in “Teacher’s role in assessment,” where constructive teachers scored lower than interpretative and reproductive ones (*p* < 0.05), indicating they tended to see GenAI as less of a threat to assessment.

Moreover, when analyzing the differences between benefits and risks of using GenAI based on pedagogical beliefs, reproductive teachers considered GenAI more of a threat to teaching and learning (*p* < 0.001), except in “Teacher’s role in teaching,” where there were no differences regarding the opportunities it offers. Interpretative teachers especially valued the threats of using GenAI for “Student’s learning processes” (*p* < 0.01), while also highlighting the opportunities GenAI offers in “Teacher’s role in teaching” (*p* < 0.001). Lastly, constructive teachers valued the opportunity that GenAI offers in all dimensions of the questionnaire (*p* < 0.05), except in “Student’s learning processes,” where they similarly valued both benefits and risks.

When analyzing the effect of previous educational use of GenAI, a significant influence (*p* < 0.001) was also observed in the differences between teachers’ beliefs about the use of GenAI as an opportunity or threat, with high interaction effect sizes (*η^2^* > 0.16). Specifically, those who used GenAI more frequently scored higher in all dimensions referring to the opportunities offered by GenAI compared to those who used it less frequently (*p* < 0.001). Conversely, those who used GenAI less frequently attached higher value to the threats in all dimensions of the questionnaire than those who used it more frequently (*p* < 0.001).

Furthermore, analyzing the differences between dimensions referring to the opportunities and threats of GenAI, it was found that those who never used GenAI always attached greater importance to the risks of its use, except in “Teacher’s role in teaching,” where there were no differences between the scales. Additionally, those who used GenAI sporadically valued the opportunities of GenAI more in “Teacher’s role in assessment” and “Teacher’s role in teaching” (*p* < 0.05), while there were no differences between the opportunities and threats of these resources in the “Student’s learning processes” and “Student’s information management” dimensions. Finally, those who used GenAI more frequently valued all dimensions related to the opportunities of GenAI more compared to the potential threats of its use (*p* < 0.001).

## 5 Discussion

Among the most notable results of our study, we can highlight that university teachers identify the possibilities and risks of GenAI similarly. However, despite these differences being small, they varied significantly based on pedagogical beliefs and previous educational use of GenAI.

It was found that pedagogical beliefs have a significant effect on teachers’ beliefs about the use of GenAI, especially in dimensions related to the opportunities offered. Teachers with more reproductive beliefs were more critical than those with more interpretative and constructive beliefs regarding the possibilities of GenAI. This finding is consistent with previous studies suggesting that constructive beliefs are associated with greater integration of GenAI in the classroom (Cabero-Almenara et al., 2024; Choi et al., 2023) and educational technologies in general (Ertmer et al., 2015; Tondeur et al., 2017).

Previous educational use of GenAI also showed a significant effect on teachers’ beliefs. Those who have never used GenAI tend to highlight more its risks or threats to learning, while those who use it more frequently have more optimistic beliefs, emphasizing its educational opportunities. This pattern highlights the importance of familiarity and experience in adopting new technologies. Exposure and training in the use of GenAI seem to reduce barriers and increase confidence in its educational applications, a finding in line with results obtained by Fietta et al. (2022) and Schepman and Rodway (2020).

When analyzing specific dimensions of beliefs, it was observed that teachers value the educational opportunities offered by GenAI more in the “Teacher’s role in teaching” dimension compared to other dimensions. This could indicate that teachers see greater potential in GenAI to support the planning and execution of educational activities tailored to students’ interests and levels. Similarly, it was identified that teachers did not consider the risks associated with the use of GenAI in the “Teacher’s role in teaching” dimension, suggesting that they dismiss the idea that GenAI could actually replace them in their teaching roles. So far, there has been advocacy for a guiding teacher role, replacing the traditional knowledge-transmitter teacher role, which has predominated in education. However, recently, GenAI seems capable of scaffolding students’ learning processes in completing tasks (Bond et al., 2024; OpenAI, 2024). But can GenAI be considered a good guide in this learning process? If the answer is yes, then what should the teacher’s role be? Here we believe it is important for educational research to work toward answering these questions.

In contrast, the “Student’s learning processes” dimension reflected the most negative attitudes toward the use of GenAI, highlighting its educational risks. This skepticism may be related to concerns about the superficiality of learning and excessive dependence on technology, which could limit the autonomous and critical development of students (Darvishi et al., 2024).

We also analyzed the effect of other variables commonly highlighted in the scientific literature that can affect beliefs about the use of ICT and, in some cases, GenAI. Contrary to some expectations based on the literature, the study found no significant differences in beliefs about GenAI based on gender, teaching experience, or area of knowledge. This could suggest that, in the specific context of GenAI, these variables do not play as determining a role as pedagogical beliefs and direct experience with these resources. However, it is possible that future studies with larger samples or in different educational contexts may yield different results.

### 5.1 Limitations and future research

This study has several limitations. Firstly, the measurement of beliefs about the use of GenAI in teaching and learning was based on self-report, which may be subject to social desirability bias. Additionally, the fact that a self-report was used implies that it is not possible to establish the directionality of the effects since all variables were collected at the same time without any intervention in the study variables.

Moreover, the obtained results are based on beliefs about the use of GenAI and not on the actual practices of these teachers. Many studies on teacher beliefs and practices have shown that the activities teachers report doing with ICT are often more frequent, complex, and varied than what is actually observed in their practices (Arancibia et al., 2020; De Aldama and Pozo, 2016; Du Plessis, 2016; Kaymakamoglu, 2018). Therefore, we believe it is necessary to investigate the relationship between beliefs and actual practices in the use of GenAI and its relationship with the variables studied in this research.

On the other hand, a potential limitation of this study, particularly in a field with limited empirical research, is the question of whether its findings can be generalized to other contexts. This is due to the sample being drawn exclusively from Spain. Future research will be necessary to replicate these findings in different countries and contexts, such as with teachers from other educational levels and environments.

Finally, it is also essential to conduct studies that examine students’ beliefs regarding the use of GenAI. Understanding how students perceive its potential, benefits, and risks is crucial for designing integration strategies that address their needs and expectations. Furthermore, future research could investigate whether students’ beliefs align with those of their teachers, as such alignment may influence the effectiveness of GenAI’s integration in education and promote a more cohesive and collaborative adoption process.

## 6 Conclusion

Generative artificial intelligence (GenAI) is an extremely powerful resource whose integration into our daily lives is increasingly evident. Young people are not oblivious to this reality; in fact, they are the ones who show more proactive attitudes toward the use of these technologies (Nikolenko and Astapenko, 2023). This suggests that students are more open to integrating these technologies into their learning process.

This scenario presents a significant challenge for teachers, who must adapt both to the needs of their students and to society’s demands concerning GenAI. As discussed throughout this study, GenAI presents significant advancements that can be a valuable opportunity for teaching. However, without the necessary competencies, its use can also entail risks that should not be ignored (Lo, 2023). Therefore, it is essential to promote teacher training in the use of GenAI.

Through this study, the impact of pedagogical beliefs and previous educational use of GenAI on university teachers’ perceptions of the use of these tools in the classroom has been identified. Promoting greater familiarity and training in the use of GenAI, along with a student-centered pedagogical approach, can facilitate the adoption of these resources in a way that maximizes their educational benefits and minimizes their risks.

It is essential to foster specific training for teachers that focuses not only on the technical management of GenAI tools but also on their effective integration into curriculum design and teaching methodologies. Based on the data from this study, greater use of GenAI in student-centered teaching tasks should be encouraged, and a shift in teachers’ pedagogical beliefs should be promoted. This shift involves moving from traditional approaches to more constructivist approaches, where students become active agents in their learning process, using GenAI as a tool that facilitates exploration, analysis, and the generation of new knowledge.

## Data Availability

The raw data supporting the conclusions of this article will be made available by the authors, without undue reservation.

## Footnotes

^1^DM, Difference of means.
