# Supplementary material for: University teachers’ beliefs about the use of generative artificial intelligence for teaching and learning
Source: Front Psychol. 2024 Dec 17;15:1468900. doi: 10.3389/fpsyg.2024.1468900 (PMC11685114; doi:10.3389/fpsyg.2024.1468900)
Supplement: Supplementary file 1 [file Table_1.docx]

**Appendix I.** *Questionnaire on Pedagogical Beliefs (De Vries et al., 2014, translated by Arancibia et al., 2020)*

| Dimensions | Items |
| --- | --- |
| Behaviorist Pedagogical Beliefs | Transmit the subject content to the students. |
|  | Ensure that the content of my classes is good. |
|  | Students acquire knowledge. |
|  | Students really listen to what I say. |
|  | There is order and discipline during the class. |
|  | Students learn the content of the subject I teach. |
|  | Students understand how to achieve better learning in the subject I teach. |
| Constructivist Pedagogical Beliefs | Students learn to solve problems related to the subject I teach independently. |
|  | Students, where relevant, learn cooperatively in work groups. |
|  | Students develop their abilities and competencies. |
|  | Establish communication with the students' own knowledge and experiences. |
|  | Consider differences in aptitudes and interests among students. |
|  | Integrate the latest advances in my curricular discipline into my classes. |
|  | Students work actively with the material I teach. |
