# Supplementary material for: University teachers’ beliefs about the use of generative artificial intelligence for teaching and learning
Source: Front Psychol. 2024 Dec 17;15:1468900. doi: 10.3389/fpsyg.2024.1468900 (PMC11685114; doi:10.3389/fpsyg.2024.1468900)
Supplement: Supplementary file 2 [file Table_2.docx]

**Appendix II.** *Questionnaire on Beliefs about the Use of GenAI for Teaching and Learning*

|  | To what extent do you believe GenAI can... | |
| --- | --- | --- |
|  | Item on the use of generative AI seen as an opportunity | Item on the use of generative AI seen as a threat |
| Student´s learning processes | …promote deeper learning by helping students better understand the content | ...hinder learning because it makes students learn more superficially, repeating GenAI's answers without trying to understand them. |
|  | ...promote a more active role for students in their learning process by requiring them to think about the questions/prompts they must ask. | ...promote a more passive role by providing students with pre-made answers without requiring them to generate them themselves. |
|  | ...allow students to learn more about a topic and give it personal meaning. | ...make students accustomed to finding quick and easy answers on a topic without much personal elaboration. |
|  | ...promote creativity by enabling students to generate new ideas they hadn't thought of before. | …limit students' creativity by generating finished answers to the requested topic. |
| Student´s information management | ...be an opportunity for students to recognize and differentiate the large amount of false or invented information it generates. | ...be a risk by containing unreliable or outdated information that can confuse students. |
|  | ...facilitate critical thinking by requiring students to contrast information from multiple sources and integrate diverse perspectives. | …encourage students to copy and paste information without questioning the sources. |
|  | …encourage students to learn to generate better questions for generative AI to seek information. | ...make students accustomed to finding answers without having formulated the required question for seeking information. |
|  | ...be an opportunity for students to become aware of the privacy issues associated with its use. | ...hinder students' awareness of privacy issues due to the lack of transparency. |
| Teacher´s role in assessment | ...be an opportunity for the teacher to promote activities addressing plagiarism issues. | ...make plagiarism easier, requiring teachers to use more powerful detection tools. |
|  | …promote new forms of evaluation beyond mere reproduction of knowledge. | ...make it more difficult to evaluate student performance because it will be hard to distinguish what they have developed themselves from what was generated by GenAI. |
|  | ...promote assessment focused on how students plan and carry out tasks. | ...make it necessary to return to traditional exams (e.g., pencil and paper or oral exams) to prevent copying. |
|  | ...help the teacher assess how students manage the feedback provided. | …make it difficult for teachers to evaluate how students arrived at their answers. |
| Teacher´s role in teaching | ...shift the teaching role from focusing on knowledge transmission to helping students manage it. | ...make the teaching role increasingly irrelevant as students extract knowledge directly from GenAI. |
|  | ...promote the teacher's role in teaching students how to use these resources better for learning. | …harm academic activities, making it advisable to prohibit or restrict its use as much as possible. |
|  | …allow teachers to tailor activities to the levels and interests of students. | ...make students focus on their interests, making it difficult for the teacher to determine if they have reached the required minimum level. |
|  | ...make it unnecessary to spend class time explaining the syllabus, allowing time for more novel and interactive student activities. | ...make teaching more difficult by requiring a lot of time to correct errors and biases generated by GenAI to ensure understanding of the syllabus. |
